# Evidence for the Role of B Cells and Immunoglobulins in the Pathogenesis of Multiple Sclerosis

**DOI:** 10.1155/2011/780712

**Published:** 2011-09-22

**Authors:** Bharath Wootla, Aleksandar Denic, B. Mark Keegan, Jeffrey L. Winters, David Astapenko, Arthur E. Warrington, Allan J. Bieber, Moses Rodriguez

**Affiliations:** ^1^Department of Neurology, Mayo Clinic, 200 First Street SW, Rochester, MN 55905, USA; ^2^Department of Laboratory Medicine and Pathology, Mayo Clinic, 200 First Street SW, Rochester, MN 55905, USA; ^3^Department of Histology and Embryology, Charles University in Prague, Faculty of Medicine in Hradec Králové, Simkova 870, P.O. Box 38, 500 38 Hradec Králové, Czech Republic; ^4^Department of Immunology, Mayo Clinic, 200 First Street SW, Rochester, MN 55905, USA

## Abstract

The pathogenesis of multiple sclerosis (MS) remains elusive. Recent reports advocate greater involvement of B cells and immunoglobulins in the initiation and propagation of MS lesions at different stages of their ontogeny. The key role of B cells and immunoglobulins in pathogenesis was initially identified by studies in which patients whose fulminant attacks of demyelination did not respond to steroids experienced remarkable functional improvement following plasma exchange. The positive response to Rituximab in Phase II clinical trials of relapsing-remitting MS confirms the role of B cells. The critical question is how B cells contribute to MS. In this paper, we discuss both the deleterious and the beneficial roles of B cells and immunoglobulins in MS lesions. We provide alternative hypotheses to explain both damaging and protective antibody responses.

## 1. Introduction

Multiple sclerosis (MS) is a chronic inflammatory demyelinating disease of the central nervous system (CNS) with varied clinical presentations and heterogeneous histopathological features. According to the National MS Society, approximately 400,000 people have been diagnosed with MS in the United States, with approximately 200 new cases every week. The main pathologic hallmark of MS is the demyelinated plaque, which has specific histological and immunocytological characteristics depending on the activity of the disease [1–4]. Another important immunopathological feature of MS is continuous synthesis of immunoglobulins (oligoclonal IgG's) in cerebrospinal fluid (CSF). The evidence associating antibodies with MS derives from a study by Kabat et al., who first described increased levels of immunoglobulin (Ig) in the cerebrospinal fluid (CSF) [5]. For many years, self-reactive antibodies have been associated with the pathogenesis of MS, and their presence was usually linked to demyelination based on studies done in experimental autoimmune encephalomyelitis (EAE), the most commonly studied model of MS [6].

EAE is induced in animals by intradermal injection with whole or parts of various proteins that make up myelin. It is primordial to understand that EAE is not MS; it only resembles some forms and stages of MS. Hence, a number of significant assumptions made when proposing EAE as MS may only be partially true. Evidence from EAE studies favoring the role of antibodies in demyelination derived from three studies, (i) serum from animals with EAE produced periventricular demyelinating lesions when injected directly into the ventricles of normal animals, [7, 8], (ii) gamma-globulin was deposited in the CNS before the appearance of cells [9]; and (iii) serum from animals with EAE produced by sensitization with whole CNS-induced demyelination of CNS tissue cultures [10]. A feature that distinguishes species is the structural and functional heterogeneity of antibodies in higher species such as mammals. EAE is commonly studied in rodents and is mostly dependent on cellular immune responses even if antibodies worsen the disease, with the exception of models where pathogenic Th2 responses play a pivotal role. The reader should understand that the heterogeneity of antibody response and the diversity in their pathogenicity was mostly suggested and best studied in primate models of EAE [11] that closely reflect adequate complexity found in humans. Two other animal models of MS are virus-induced or toxin-induced demyelination [6]. Using a mouse model of Theiler's virus-induced demyelination, our research group showed that immunoglobulins and complement are involved in the pathogenesis of demyelination [12]. In a later study, in humans, we reported on four different pathologic subtypes of active MS lesions among 83 biopsies and autopsies of MS patients [1]. One of the subtypes, Type II, demonstrated a prominent presence of antibodies and complement. Antibodies directed against self-antigens are frequently considered deleterious and may play a pathogenic role in certain autoimmune diseases. A conventional method to remove circulating autoantibodies in patients is termed plasma exchange (PLEX). PLEX is a procedure that involves separating the blood from plasma and exchanging the plasma with albumin and returning the cellular components back to the patient. The large molecular weight substances that are removed from plasma following PLEX remain a question of open debate although it is certain that immunoglobulins and immune complexes are a major component of the substances that are removed. Few studies described evidence that PLEX does more than just remove antibodies; PLEX (i) can alter immune system by affecting T helper type-1/T helper type-II balance of circulating peripheral lymphocytes [13], (ii) can shift Th2-dominant status to Th1-dominant status [14], (iii) can decrease cytokine levels [15], and (iv) can improve cell function when administered along with prednisone [16]. 

## 2. Effect of Plasma Exchange in Patients with Demyelinating Disorders

Our group demonstrated conclusively for the first time the detrimental effect of plasma components in inflammatory demyelinating diseases of the CNS; PLEX in acute episodes of fulminant CNS inflammatory demyelination, which did not respond to high-dose methylprednisolone, led to a marked neurologic improvement in 6 patients [18]. These results were later confirmed in a double-blind placebo-controlled (sham procedure) trial performed at the Mayo Clinic, which demonstrated that approximately 40% of patients with acute, severe neurological deficits caused by MS or other CNS inflammatory demyelinating diseases who failed to recover after treatment with high-dose corticosteroids have dramatic recovery following PLEX [19]. In other well-designed, controlled studies, the beneficial effect of PLEX was demonstrated in patients with MS [20–22]. A more recent study [23] from our group performed in 153 patients with acute, steroid-refractory CNS inflammatory demyelinating disease (CNS-IDD) demonstrated moderate to marked functional neurological improvement in 59% of the cases within 6 months following PLEX. However, PLEX was less effective for patients with multiple sclerosis who subsequently developed a progressive disease course (*P* = .046). Accordingly, a recently published evidence-based guideline update concluded that PLEX is (i) probably effective and should be considered as a second-line treatment of steroid-resistant exacerbations in relapsing forms of MS, (ii) possibly effective and may be considered for acute fulminant demyelinating CNS disease, and (iii) is ineffective and should not be considered for chronic or secondary progressive MS [24]. Treatment in relapsing-remitting MS includes azathioprine, intravenous immunoglobulin (IVIg), interferon *β*-1a, glatiramer acetate, mitoxantrone hydrochloride, natalizumab, and cyclophosphamide, depending on the disease severity. A randomized, double-blinded, placebo-controlled trial of IVIg in patients with MS who had persistent muscle weakness concluded that IVIg does not reverse established weakness in MS [25]. PLEX has not been specifically studied in relapsing-remitting MS. A traditional hampering factor remains to be the excessive cost burden on the patient to undergo PLEX procedure. Nevertheless, a recent estimate established that a direct cost of five IVIg infusion sessions totaling 2.0 grams per kilogram (g/kg) body weight was $10,329.85 compared to a series of five TPE procedures, which had direct costs of $4,638.16 [26]. Taking this into consideration, further studies to understand the beneficial effects of PLEX are warranted given that the optimal protocol and the duration of benefit remains un-established. 

## 3. The Presence of B Cells in CNS

An MS plaque is histologically characterized by inflammation, demyelination, and gliosis. Infiltration by mononuclear cells, particularly macrophages and T cells, is typical of the acute MS lesion. Esiri showed that autopsy material from the brains and spinal cords of 23 MS patients showed that Ig-containing cells were more numerous within the plaques than outside and were more common in recent than in old plaques [27]. Subsequently, Cepok et al. showed that B cells account for up to 25% of the CSF-infiltrating leukocytes during CNS inflammatory responses [28]. To better understand the potential mechanism of B cells in MS pathology, Baranzini et al. had PCR amplified and analyzed by size, spectratyping, and sequencing the Ig heavy chain CDR3 repertoire in 10 MS brain samples. Their work showed a higher level of rearranged transcripts in MS brains when compared to the noninflammatory brain tissues, suggesting that oligoclonal bands found by spectratyping are the result of antibody synthesis within the plaques [29]. The above findings supported the hypothesis that a germinal center- (GC-) like reaction takes place during the immune attack against CNS structures. This concept was further strengthened by Colombo et al. [30] who detected oligoclonal B cell accumulations in 10 of 10 MS patients. Upon analyses of the Ig V(D)J sequences from the CSF of patients, it was found that variable heavy chain domains, V_H_3 and V_H_4 genes, were extensively mutated as compared to the germline sequences. Their data suggested a compartmentalized clonal expansion in MS. This, however, raised the question on the capability of the brain tissue to host a GC-like reaction. The works of Knopf et al. provided the answers: they used a rodent model with an intact blood-brain barrier and demonstrated the trafficking of activated antigen-specific B cells into the brain, retention, and antibody production [31]. Their data proved that the brain microenvironment supports the development of antigen-directed humoral immunity. To confirm this concept, Corcione et al., showed that each B-cell subset participating in the GC reaction could be detected in the CSF of MS patients [32]. 

## 4. Autoimmune Hypothesis and the Presence of Immunoglobulins in CNS

Autoimmunity is the most accepted theory for MS pathogenesis, as self-reactive antibodies have been implicated in the pathogenesis of MS. Previous studies suggested intrathecal production of antibodies occurred after clonal expansion in patients as seen by the consistent identification of oligoclonal bands after CSF electrophoresis [33]. We reported on the heterogeneity of MS lesions in CNS tissue and their implications for the pathogenesis of demyelination. We performed a detailed immunohistochemical study of active MS lesions from 83 biopsies and autopsies of MS patients, following which we identified four different pathologic subtypes of active MS lesions. One of the subtypes, Type II, demonstrated the presence of macrophages and T-cells and, in addition, a prominent display of antibodies and complement [1]. Numerous publications during the last few decades supported the idea that CSF oligoclonal bands correlate to the level of B-cell involvement in MS [34]. In addition, evidence indicates that oligoclonal bands may have a prognostic value. One prospective study of patients with acute isolated demyelinating episode demonstrated intrathecal immunoglobulin synthesis to be a better predictor of MS progression than MRI [35]. Another prospective study showed that the presence of CSF oligoclonal bands in early MS generally correlated to a worse outcome [36]. A recent study showed strong correlation between levels of oligoclonal bands (OCBs) and prognosis for MS disability [37]. 

Our work [1, 18, 19] and others' results [38, 39] favor antibody-mediated demyelination in MS. On the contrary, in a recent study comparing the immunoglobulins and activated complement in MS and other neurologic diseases (ONDs) patients, the authors did not find IgG in myelin or ramified microglia from tissue remote to focal lesions [40]. Interestingly, C3d and C9neo stained positively for all IgG from MS and ONDs. The only MS-specific IgG deposition they found consisted of unusual microglial nodules containing short, linear deposits of activated complement (C3d) on partially demyelinated axons located in normal-appearing periplaque white matter. This study thus argued that the IgG and complement immunostaining of disrupted myelin in MS lesions is a nonspecific feature that cannot be interpreted as evidence of a distinct pathogenesis or serve to define particular variants of the disease. However, it is questionable whether many of the lesions in the study were as acute as those in the studies performed at Mayo [1].

In the past, Avrameas proposed that autoreactive antibodies present in healthy humans have natural physiological roles [41]. In line with this, we have shown that immunoglobulins directed against antigens of the lipid rafts of oligodendrocytes promote remyelination in CNS. Systemic injection of serum molecules from donor mice hyperimmunized with homogenized spinal cord induced the remyelination of CNS axons. This was the first evidence that immunoglobulins secreted in demyelinating lesions may have the potential to promote myelin repair [42]. We have further demonstrated that human immunoglobulins, when used to treat animal models of disease, can promote remyelination [43] and neurite extension [44]. Protecting axons of the CNS promises to be an effective strategy to limit axon loss and prevent permanent disability. Thus, there is a delicate balance between the presence of beneficial and deleterious immunoglobulins at the site of active lesion. 

## 5. Antigen Specificity of Autoantibodies Found in MS

After several years of research, confirmation of the antigen-specificity of autoreactive antibodies in MS is still lacking. Due to their broad reactivity, IgG in CSF of patients with MS may represent synthesis of “nonsense” antibodies irrelevant to pathogenesis [45–47]. However, other experiments found molecular uniformity and temporal persistence of the Ig response in MS, thus conflicting with the nonsense antibody proposal [48]. It is possible that relevant molecules are limited to the myelin sheath. Warren and Catz studied the specificities of autoantibodies from MS patients and reported that most patients' IgG bound to MBP. They also reported that the peptide MBP [49–63] strongly inhibited autoantibody binding to MBP in almost all cases tested [64]. Genain et al. offered the most convincing evidence. Using immunogold-labeled peptides of myelin antigens and high-resolution microscopy (techniques that can detect antigen-specific antibodies *in situ*), they identified autoantibodies specific for the CNS myelin antigen myelin/oligodendrocyte glycoprotein (MOG). These autoantibodies specifically bound to disintegrating myelin around axons in acute MS lesions and the marmoset model of EAE [65]. O'Connor et al. examined whether autoantibodies that bind properly folded MOG protein are present in the CNS parenchyma of MS patients. Their data demonstrated that MOG-recognizing autoantibodies are present in substantially higher concentrations in the CNS parenchyma of MS patients than in the CSF and serum. This indicated that local production/accumulation is an important aspect of autoantibody-mediated pathology in CNS demyelinating diseases [66]. Further studies demonstrated the serological and/or CSF presence of antibodies directed against MBP and/or MOG in patients with MS [67]. 

Conversely, myelin-specific antibodies are not limited to MS. Using an enzyme-linked immunosorbent assay, Karni et al. compared levels and frequencies of anti-MOG antibody between patients with MS, patients with ONDs and healthy control subjects. Interestingly, the authors found higher plasma levels of antibodies to MOG and to MBP in MS patients compared with ONDs patients; however, the frequency of antibodies to MOG and MBP was similar in MS, ONDs and healthy controls [68]. Another group presented analogous results. Lampasona et al. used a liquid-phase radiobinding assay to measure serum anti-MOG IgG among 87 MS patients with MS, 12 encephalomyelitis patients and 47 healthy subjects. Surprisingly, the frequency of positive samples with low titers of anti-MOG IgG was similar in all the groups and subgroups. Binding-competition experiments showed that low affinity in these antibodies. These results demonstrated that anti-MOG antibodies are not disease specific [69]. 

In a parallel line of research, some reports suggested lipids or carbohydrates as possible candidate antigens for the humoral immune response. Arnon et al. reported the presence of anti-ganglioside antibodies in MS [70]. Endo et al. reported the presence of antibodies to glycosphingolipids in MS patients [71]. Their results indicated that antibodies against ganglioside GM1 and asialo GM1 were found commonly in 34 of 46 patients with MS. However, antilipid antibodies can be found in other diseases, such as systemic lupus erythematosus and stroke [72, 73]. An interesting report identified anti-alpha-glucose-based glycan IgM antibodies as predictors of relapse activity in MS after the first neurological event [74]. Others suggested that serum anti-Glc(alpha1, 4)Glc(alpha) antibodies serve as biomarkers for relapsing—remitting MS [75]. Villar et al. reported that the intrathecal synthesis of oligoclonal IgM against myelin lipids may predict an aggressive disease course in MS [76]. The most frequently recognized lipid in their study was phosphatidylcholine. Autoantibodies to myelin proteins, lipids and carbohydrates can be extracted from the tissue and sera of some MS patients. 

A major discovery in demyelinating disorders was when Hinson et al. [77–79] discovered a potential pathogenic immunoglobulin G binding to the extracellular domain of a water channel aquaporin-4 (AQP4) in patients with neuromyelitis optica (NMO). Potential pathogenic immunoglobulins appear in approximately 70% of patients with NMO. It is unknown whether a detection problem limits the assay in the remaining patients or if an autoantibody to a different antigen drives the response. However, no clinical differences exist between antibody-positive and antibody-negative patients with clinical NMO. A randomized trial to determine whether IVIg reverses chronic visual impairment in MS patients with optic neuritis (ON) demonstrated that IVIg administration does not reverse persistent visual loss from ON to a degree that merits general use [80]. Interestingly, PLEX is a highly successful treatment for NMO arguing in favor of an autoimmune-mediated pathogenesis of this disease. In line with the autoimmune-mediated hypothesis, humoral immunity-suppressing drugs such as Mitoxantrone hydrochloride [81], (a synthetic anthracenedione that was approved for the treatment of worsening relapsing-remitting and secondary progressive MS), Mycophenolate Mofetil [82], (an immunosuppressive therapy), and Rituximab [83], (a B cell depleting therapy) were demonstrated to be beneficial for treatment of NMO. 

It is now considered that NMO is likely the first true demyelinating autoimmune disorder. Autoantibodies may contribute to the ongoing immune response, but after more than 50 years of investigation in the field, one still cannot substantiate all the criteria necessary to classify MS as an autoimmune disease [84]. In light of this, the autoimmunehypothesis requires a complementary hypothesis/reason for pathogenesis of MS.

## 6. Alternate Hypothesis of MS Pathogenesis

MS is a complex and heterogeneous disease; hence, in addition to the autoimmune hypothesis, it is assumed that certain infectious agents play an important role in the pathogenesis. Epstein-Barr virus (EBV), human herpes virus-6 (HHV-6), varicella zoster virus (VZV), and Chlamydia pneumonia are some of the proposed infectious agents. Many studies in this area have demonstrated the presence of antibody titers to a broad range of pathogens in MS patients; however, many of these findings remain solitary and unconfirmed by other groups. EBV is a B-lymphotropic human DNA herpes virus that infects most individuals asymptomatically but causes infectious mononucleosis (IM) in some [85, 86]. Cepok et al. identified EBV proteins as putative targets of the immune response in MS [87]. Another study demonstrated the increased risk of MS in individuals with a clinical history of IM [88, 89]. Recently, researchers from the United Kingdom studied the prevalence of MS and infectious mononucleosis (IM) and how they relate to ultraviolet B (UVB) exposure [90]. As previously shown in other studies, MS highly correlated with IM [91, 92]. As a control, the authors also examined correlations of MS with cytomegalovirus prevalence and varicella prevalence, respectively, both of which were nonsignificant. Of note, the authors found that UVB in any season correlated more closely with MS than with IM. These results fit well with the EBV hypothesis, because there may be a mechanism through which UVB radiation mediates MS risk. 

**Figure 1:**
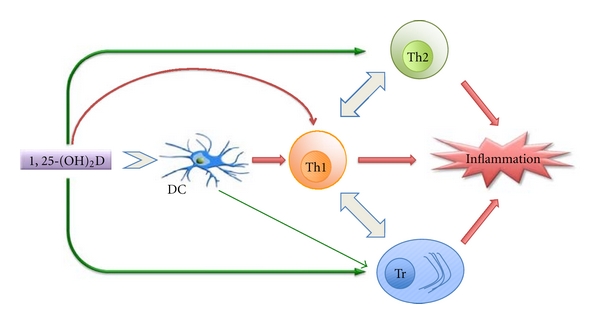
The *in vitro* effects of 1,25(OH)_2_D on the immune system. The effects of 1,25(OH)_2_D either directly or indirectly are depicted by arrows. While a green arrow represents positive influence, a red arrow represents the negative influence. The negative influence on inflammation indicates dampening of the inflammatory response. DC: dendritic cell, Th1: T helper type 1 lymphocyte, Th2: T helper type 2 lymphocyte, Tr: regulatory T lymphocyte [17].

It has been suggested that low vitamin D levels as a result of immunosuppression lead to an increase in EBV infection. It is also known that a low amount of UVB decreases vitamin D levels. The geographical variation in the MS prevalence, with a higher prevalence of the disease in northern latitudes and a lower prevalence at the equator, is well established [93–95]. This variation in MS prevalence correlates positively with changes in the serum concentrations of 25-hydroxyvitamin D [96–98]. Several, but not all, studies show an inverse correlation between serum 25-hydroxyvitamin D concentrations and the incidence of MS, the severity and progression of disease [49–61]. Vitamin D, and its biologically active metabolite 1,25-dihydroxyvitamin D_3 _(1,25(OH)_2_D_3_), not only plays an important role in the regulation of calcium and phosphorus homeostasis, but also is an important modulator of immune function. 1,25(OH)_2_D_3_ functions by associating with the vitamin D receptor that is widely distributed in a number of calcium-transporting tissues, neural tissues, and immune cells (dendritic cells, T-lymphocytes, B-lymphocytes, and macrophages) [62, 63, 99–104]. 1,25(OH)_2_D_3_ increases macrophage activity, inhibits dendritic cell maturation, inhibits B-cell functions, and favors the production of T-helper2 cells, thereby shifting the ratio of Th1/Th2 cells in favor of Th2 helper cells (Figure 1) [17, 105–111]. The polarization of activated CD4 + T-cells to a Th-1 phenotype (IL-2, IFN*γ*, TNF*α* and secretion) or to a Th-2 phenotype (IL-4, 5, 13, and 10 secretion) represents a major determinant of the nature of subsequent cellular and humoral immune responses. It is a self-perpetuating process in that one subtype inhibits the generation of the other [108, 110]. The primary generation of Th-1-type T-cell responses is potently inhibited by 1,25(OH)_2_D_3_ both *in vitro* and *in vivo*. 1,25(OH)_2_D_3_ also induces production of human cathelicidin, LL-37, which is particularly effective against respiratory viruses such as influenza [112]. The lack of vitamin D may result in abnormal response to EBV infection causing IM, thereby leading to a higher risk for MS [113]. 

## 7. Role of B Cells

### 7.1. As Antigen-Presenting Cells

The role of B cells in acute demyelination was discussed in the previous sections (accumulation of clonally expanded B cells, neuropathological studies, and presence of complement). Besides their role in acute demyelination, B cells can contribute to the disease progression in MS [114]. It was previously shown that epitope specificities generated in a mouse model of MS, EAE, overlap with encephalitogenic T cell epitopes, and human immune-dominant T and B cell epitopes [115–117]. Harp et al. studied the impact of myelin-specific antigen-presenting B cells on T cell activation in MS. Their results demonstrated a possible role of B cells as myelin-specific antigen-presenting cells (B-APCs) in MS [118]. 

Another treatment strategy used is Rituximab, an anti-CD20 monoclonal antibody. CD20 is a surface antigen with restricted expression on pre-B and mature B cells, but it is not expressed on stem cells and cells differentiating into plasma cells. Recent reports showed some favorable results of Rituximab in relapsing-remitting MS patients, as the administration of this drug reduced inflammatory brain MRI lesions and clinical relapses for 48 weeks in patients, after a single dose course [119, 120]. A study by Cross et al. showed that Rituximab reduces B and T cells in CSF of MS patients [121]. However, the rapid effect of Rituximab is not explained by the fact that it does not act on plasma cells or cells differentiating into plasma cells. Rituximab may target the processes such as antigen presentation by B cells and activation of T cells. This may affect the production of proinflammatory and regulatory cytokines in the CNS microenvironment. In line with this, a recent study in MS patients on Rituximab demonstrated that the episodic triggering of abnormal B-cell cytokine responses mediated “bystander activation” of disease-relevant proinflammatory T cells [122].

### 7.2. As Cytokine-Producing Cells

As described before, the study of Bar-Or et al. proposed that abnormal B-cell cytokine responses were responsible for new MS relapses through bystander activation of relevant proinflammatory T-cells [122]. This finding was important and may explain the established association between new MS relapses and infections. The importance of balanced proinflammatory and anti-inflammatory B-cell cytokines was demonstrated when selective deletion of IL-10 from B-cells aggravated clinical disease in EAE [123]. In addition, a recent study showed that IL-10 secretion by B-cells was deficient in MS patients as compared to healthy controls [124]. However, levels of IL-10 production were equivalent in patients with relapsing-remitting and secondary progressive MS. 

## 8. Mechanisms of Action of MS Antibodies

Antibodies are the antigen-binding proteins present on the B-cell membrane and are secreted by the plasma cells. Membrane-bound antibody confers antigenic specificity on B cells; antigen-specific proliferation of B-cell clones is elicited by the interaction of membrane antibody with antigen. The versatility of secreted antibodies is demonstrated by the functions they mediate such as neutralization, agglutination, fixation with activation of complement, and activation of effector cells. Among the antibody functions is a novel property of the antibodies that fascinated scientists for several years. It is the ability of some antibodies to behave as enzymes with catalytic ability. Catalytic immunoglobulins, both of the IgM and IgG isotypes, have been detected in the serum of healthy donors, where they presumably participate in removing metabolic wastes and defend the organism against invading pathogens. Conversely, antigen-specific hydrolytic IgGs are seen in a number of inflammatory, autoimmune, and neoplastic disorders; their pathogenic effects have been demonstrated occasionally. Studies during the past decades have shown antibodies to be capable of participating in at least one of the functions presented below.

### 8.1. Functions of Immunoglobulins

**(1) Neutralization**

The Fab region of the antibodies binds to the target (viruses, microbes [125], and/or toxins [126]) and blocks or neutralizes their action. There is some evidence that antibodies present in plaques bind to MBP and/or MOG and neutralize and cause deterioration of the tissue; however, the results remain controversial due to the presence of antibodies with similar specificities in healthy blood donors.

**(2) Agglutination**

Antibodies are clonally generated for binding single specific antigens. The Fab regions of the antibodies link the antigens together, causing them to clump together, also known as agglutination [125]. Monoclonal antibodies derived from MS patients' agglutinated liposomes made from lipids of a chloroform/methanol extract of human myelin. Investigations by ELISA suggest that phospholipids are the reactive components, at least for some of these mAbs. Some antibodies reacted with liposomes containing galactocerebroside or sulfatide, others only with sulfatide-containing liposomes. However, there is no reported evidence for role of this binding for MS [127].

**(3) Fixation and Activation of Complement**

The antibodies that bind to surface antigens on, for example, a macromolecular structure, will attract the first component of the complement cascade with their Fc region and initiate the activation of the “classical” complement system [128]. This results in the destruction of the molecule [125]. We previously described complement deposition in MS plaques [1]. Complement activation is known to occur in white-matter MS lesions. Storch et al. described a case of MS characterized by deposition of immunoglobulin and complement in the areas of active demyelination. In addition, macrophages in the lesions contained degradation products that were immunoreactive for myelin antigens and immunoglobulins. They observed that the destruction of myelin sheaths was associated with incomplete loss of oligodendrocytes in the active areas and reappearance of oligodendrocytes with remyelination in the inactive plaque center [129]. Finally, Piddlesden et al. showed that the demyelinating potential of antibodies to MOG was related to their ability to fix complement [130].

**(4) Activation of Effector Cells**

Mast cells and phagocytes have Fc receptors that interact with the Fc region of IgA, IgG, and IgE antibodies. The engagement of a particular antibody with the Fc receptor on a particular cell triggers the effector function of that cell (e.g., phagocytes will phagocytose and mast cells degranulate) that will ultimately result in destruction of the invading microbe. One or more FcRs are expressed in microglia, astrocytes, oligodendrocytes, and neurons. Aberrant activation of FcRs in such neural cells may contribute to MS pathogenesis [131]. Antimyelin antibodies may mediate damage to myelin membranes through separate mechanisms such as receptor-mediated phagocytosis by macrophages and/or presentation of myelin autoantigens to specific T cells [65]. Autoantibodies may activate mast cells and cause their degranulation. The concept of mast cells in MS dates back to more than a century. Neumann in 1890 described the first association of mast cells to MS plaques [132]. Olsson identified mast cells in MS plaques, in 1974 [133]. Kruger et al. suggested in 1990 that mast cells contribute to the demyelinating process of MS [134]. A definite role for mast cells was finally demonstrated using the mast cell-deficient mice. These mice exhibited delayed onset and decreased EAE disease severity when compared to wild-type littermates [135].

**(5) Effector Functions of Immunoglobulins**

The Fc region mediates effector functions, such as antibody-dependent cellular cytotoxicity (ADCC) and complement-dependent cytotoxicity (CDC). In ADCC, the Fc region of an antibody binds to Fc receptors (Fc*γ*Rs) on the surface of immune effector cells such as natural killers and macrophages, leading to the phagocytosis or lysis of the targeted cells. In CDC, antibodies kill the targeted cells by triggering the complement cascade at the cell surface. Frick and Stickl demonstrated that antibodies from sera of MS patients enable normal lymphocytes to exhibit a cytotoxic reaction against MBP [136, 137]. Later, Merrill et al. compared the peripheral blood lymphocytes (PBL) of MS patients to age- and gender-matched controls for ability to mediate ADCC. They demonstrated an enhanced ADCC of blood lymphocytes of MS patients [138].

**(6) Catalytic Activity of Ig**

In most of the above-described functions of Ig, the antibodies tightly bind the antigen but does not specifically alter its chemical nature. Natural enzymes within the body bind biomolecules and subsequently catalyze their conversion to new products. Within the enzyme itself, the amino acid sequences that form the active site determine specificity and the efficiency. Considering the huge variability of the CDR regions, it is possible to find similar amino acid sequences in the hypervariable zone of the Igs, which could confer an enzymatic function to the antibody. Indeed, antibodies that catalyze a wide variety of reactions have been reported. The first description of catalytic antibodies to MBP was documented in SJL mice with EAE [139]. Since then, hydrolytic anti-MBP antibodies were isolated from MS patients [140]. Six preferential cleavage sites of the MBP molecule were identified by abzymes isolated from patients with MS [140, 141]. All the identified cleavage sites were located c-terminus to either arginine or lysine residues, thus providing an insight into the possible mechanism of action of abzymes (a serine protease-like activity). Aprotinin, a binding inhibitor of trypsin, could not block abzyme-mediated hydrolysis. The mechanism of action of these abzymes in comparison to conventional serine proteases would be of interest to protein biochemists studying structure-function relationship of proteins.

**(7) Remyelinating Antibodies**

The discovery of the function of natural autoantibodies for CNS remyelination by our group was serendipitous. At the point of discovery, we were trying to induce more demyelination in an animal model of demyelinating disease (Theiler's virus infection) to test the hypothesis of virus-induced autoimmunity. However, when we immunized animals with myelin months after Theiler's virus infection, as opposed to extensive demyelination [142], we observed extensive remyelination [42]. As a result, we performed a classical passive transfer experiment in which we transferred antisera or purified immunoglobulins from uninfected animals immunized with myelin antigens into animals with extensive chronic demyelination following Theiler's infection [143, 144]. Of interest, the animals receiving hyperimmune sera or immunoglobulin directed against myelin showed extensive remyelination in contrast to animals that received the control antisera. Our research group then identified 8 different mouse mAbs that promoted remyelination [145]. Of note, each mAb bound unique antigens or to the surface of oligodendrocytes [146]. They all had relatively conserved germ line sequences [147, 148]. Based on this observation, we asked whether natural autoantibodies were present in the human population. We sought patients with disease processes that cause them to make their own mAbs, specifically patients with multiple myeloma, Waldenstrom's syndrome and monoclonal gammopathy of unknown significance. The serum samples from these patients were screened for binding to sliced live cerebellum and, specifically, to oligodendrocytes in culture. Two mAbs that we screened, sHIgM22 and sHIgM46, demonstrated very extensive remyelination [43] in both Theiler's virus and in lysolecithin-induced demyelination [149]. We also identified antibodies (sHIgM12 and sHIgM42) that support neurite extension *in vitro* as well as in the potent substrate laminin and override the neurite outgrowth inhibition of CNS myelin [44]. sHIgM22 [150] and sHIgM12 [151] are now available as recombinant IgM molecules. These results provide strong evidence for a beneficial response of antibodies in demyelinating diseases.

## 9. Role of Immunoglobulins

### 9.1. Deleterious Role

B cells also produce myelin-specific antibodies that bind to and destroy myelin within the plaque through an enzymatic mechanism. In this respect, catalytic antibodies have been reported to play a role in site-specific myelin destruction [139, 140]. The hydrolytic activity of the anti-MBP antibodies correlated with the expanded disability status [152]. Belogurov et al. [39] reported on the recognition and degradation of MBP peptides by serum autoantibodies. The authors used a constructed MBP-derived recombinant “epitope library” that spanned the entire MBP molecule to define the epitope-binding/ cleaving activities of autoantibodies isolated from the sera of 26 MS patients and 11 healthy individuals. The levels of autoantibodies to MBP fragments, as well as to whole MBP and myelin oligodendrocyte glycoprotein (MOG) molecules, were significantly higher in the sera of MS patients than in those of healthy donors. Patients with MS (77% of progressive and 85% of relapsing-remitting) were positive for catalysis, showing pronounced epitope specificity to the encephalitogenic MBP peptide 81–103. No healthy donors presented with these characteristics. Based on the results, anti-MBP binding and cleavage by abzymes may be regarded as a specific feature of MS as compared to healthy donors and may provide an additional marker of disease progression.

### 9.2. Beneficial Role

Our group reported on the identification of monoclonal IgM antibodies with remyelinating (sHIgM22 and sHIgM46) and neuroprotective (sHIgM12 and sHIgM42) properties. We have demonstrated that rHIgM22 binds to oligodendrocytes and myelin and promotes CNS remyelination in virus- and toxin-induced models of MS. Spinal cord remyelination is induced after a single low dose (25 *μ*g/mL) of rHIgM22 [153]. It is remarkable that one peripheral (*i.p.*) treatment with a short-lived molecule (15 hr half life in mice) promotes maximal tissue repair within 5 weeks in a model of MS with little spontaneous repair. The properties of rHIgM22 are similar to a targetable growth factor. After peripheral injection, rHIgM22 crosses the blood brain barrier (BBB) and accumulates within brain and spinal cord lesions of mice with demyelination. Ferritin bead-labeled rHIgM22 has been detected in lesions *in vivo* by magnetic resonance imaging showing that the antibodies can readily cross the BBB [154]. In addition, repair-promoting antibodies induced distinct Ca^2+^ signals in both astrocytes and oligodendrocytes. The antibody's ability to induce Ca^2+^ signals is statistically correlated with promotion of myelin repair [155]. In addition, we showed that rHIgM22 strongly inhibits apoptotic signaling via reduction of caspase-3 and caspase-9 cleavage and reduces expression of differentiation markers MBP and MOG in oligodendrocyte cultures. We also documented that remyelination-promoting human IgMs protect oligodendrocytes in culture from peroxide-induced activation of caspase 3 [156], a marker of active apoptosis. Our group then identified Lyn kinase as a key player in rHIgM22-mediated effects in OLs. We isolated integrin *α*v*β*3 and PDGF*α*R in a complex together with Lyn kinase, suggesting that rHIgM22 acts through a signaling complex containing Lyn, integrin *α*v*β*3, and PDGF*α*R in oligodendrocytes. This implies that IgM-mediated remyelination is due to protection of oligodendrocyte progenitor cells and oligodendrocytes rather than promotion of OPC differentiation [157].

## 10. Conclusions

Over the past decades, accumulated evidence emphasized the role of immunoglobulins in MS. The antibodies are characterized with both detrimental as well as beneficial roles. A number of fundamental questions related to important problems of immunoglobulins in MS still have ambiguous answers. New experimental design strategies and technologies will help answer these questions more precisely. A pivotal question is why some immunoglobulins cause deleterious effects, whereas others are beneficial. Is antigen recognition a unique attribute that determines this role? This seems unlikely given that myelin-specific antibodies are reported from both MS patients and healthy controls [158]. 

We propose that their microenvironment determines whether immunoglobulins assume a pathogenic or reparative role. Antibodies play a deleterious role when bound to myelin and result in its degradation [140]. Alternatively, antimyelin antibodies clear myelin debris from sites of acute degradation to promote remyelination [159]. Another factor may be the intracellular events mediated in the target cell upon binding. We have shown that polyclonal Ig treatment strategies, such as IVIg or IVIgM, promote CNS repair via Fc-mediated activation of microglia and stimulation of IL-1*β* release. The interaction of Fc*γ* or Fc*μ* with receptors on microglia may initiate a cascade of events culminating in IL-1*β*-induced oligodendrocyte progenitor maturation and subsequent remyelination of demyelinated lesions [160]. These observations support the hypothesis that the oligoclonal immune response [27, 28, 32] found in the CSF and CNS parenchyma of MS patients with MS may represent a powerful endogenous repair system. The evidence of antimyelin antibodies in healthy donors again supports the idea that these responses are not pathogenic but may be part of an endogenous repair system. 

**Figure 2:**
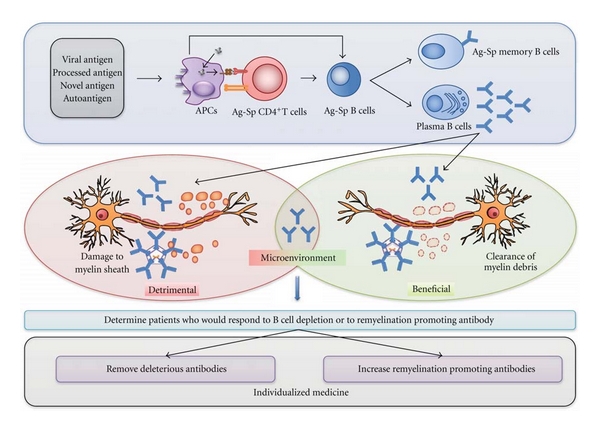
The role of B cells in MS pathogenesis. Antigens (viral, processed, novel, or auto) are internalized and presented *via* antigen presenting cells (APC's, such as dendritic cells, microglia and other mononuclear phagocyte system cells) to antigen-specific naïve T cells and/or B cells. B cells can sometimes act as APC's. Activated B cells undergo clonal expansion and mature into either antigen-specific memory B cells or into plasma cells that secrete antigen-specific antibodies that may have a detrimental (further damage to myelin sheaths of axons) or a beneficial effect (remyelination, clearing the metabolic wastes away, and neurite extension), depending on the microenvironment. The oligoclonal immune response observed in patients with MS thus represents an ambivalent role. It is of priority to determine clinical assays to delineate patients who would respond to B cell depletion therapies, or to remyelination promoting antibody therapy. This approach supports the concept of “individualized medicine”, where deleterious antibodies would be removed from circulation or in other cases endogenous remyelination would be promoted.

All approved treatments for MS have targeted T-cells. Because of the clear role of B-cells in MS pathogenesis, there is a need to develop strategies to target this cell type. Treatment of MS patients with either B cell-depleting therapies, such as the administration of Rituximab or PLEX, has had very significant positive effects on a small percentage of the MS population. However, the majority of patients show minimal response to these treatments. In light of these findings, we consider it imperative to develop *in vitro* laboratory assays to distinguish, which patients respond to strategies to deplete B cells or remove immunoglobulins. Similarly, it will be critical to determine which patients respond to a human monoclonal antibody to promote remyelination. The results from these assays can be used to select or optimize patient's preventive or therapeutic care. This is especially important for patients with progressive forms of MS or patients who have not responded to any currently available treatments. For instance, PLEX could be considered as a first line of treatment for patients with injurious or deleterious immunoglobulins. Conversely, in patients with axonal preservation, an endogenous remyelination-enhancing approach may be appropriate. Ultimately, this supports the concept of “individualized medicine” (Figure 2). However, the paucity of available data from serology, genomics, proteomics, and metabolomics from large cohorts of MS patients remains a major limitation.
